# Influence of Cement Thickness, Dentine Thickness, and Intracoronal Depth on the Fracture Resistance of 3D-Printed Endocrowns: A Pilot In Vitro Study

**DOI:** 10.3390/dj13060263

**Published:** 2025-06-12

**Authors:** Osama Abuabboud, Adrian-George Marinescu, Mihai Paven, Izabella-Maria Kovacs, Luminita Maria Nica, Andrei-Bogdan Faur, Dan Ioan Stoia, Anca Jivănescu

**Affiliations:** 1 Department of Restorative Dentistry and Endodontics, TADERP Research Center, Faculty of Dentistry, “Victor Babeș” University of Medicine and Pharmacy, 300041 Timișoara, Romania; marinescu.adrian@umft.ro (A.-G.M.); mihai.paven@umft.ro (M.P.); izabella.kovacs@umft.ro (I.-M.K.); nica.luminita@umft.ro (L.M.N.); 2 Department of Prosthodontics, TADERP Research Center, University of Medicine and Pharmacy “Victor Babeș”, B-dul Revoluției 1989, No. 9, 300580 Timișoara, Romania; andrei.faur@umft.ro (A.-B.F.); jivanescu.anca@umft.ro (A.J.); 3 Department of Mechanics and Strength of Materials, Politehnica University Timișoara, 300006 Timișoara, Romania

**Keywords:** 3D-printed endocrowns, adhesive interface, cement thickness, dentine thickness, digital dentistry, fracture resistance, prosthetic restorations

## Abstract

**Background/Objectives:** Endodontically treated molars are structurally weakened due to internal tissue loss, increasing their risk of fracture. Endocrowns, developed as a conservative alternative to post–core systems, have gained popularity with the rise of digital dentistry, CAD/CAM workflows, and 3D-printed restorations. In this context, the aim of the present pilot study was to investigate the influence of cement layer thickness, intracoronal depth, and dentine wall thickness on the fracture resistance of molars restored with 3D-printed endocrowns. **Methods:** Twelve extracted human molars were endodontically treated and restored with endocrowns fabricated from a 3D-printed resin material, SprintRay Crown^TM^ (SprintRay Inc. Los Angeles, CA, USA), via masked stereolithography (MSLA) on a Prusa SL1 printer. Cementation was performed using RelyX Universal Resin Cement (3M, Maplewood, MN USA). Cone beam computed tomography (CBCT) was used to measure the dentine thickness and intracoronal depth before cementation and cement thickness after cementation. The fracture resistance was evaluated using a universal testing machine. For each variable (Td, Dp, Tc), the 12 specimens were divided into two groups (*n* = 6). Statistical analysis included Pearson correlation, a one-way ANOVA, and the non-parametric Mann–Whitney U test. **Results:** Within the limitations of this pilot in vitro study, cement thickness demonstrated a strong positive correlation with fracture resistance (r = 0.577) and was the only variable showing statistical significance in the ANOVA (F = 7.847, *p* = 0.019). In contrast, intracoronal depth and dentine wall thickness exhibited weaker and nonsignificant correlations. No significant mechanical advantage was observed from increasing the pulp chamber depth or peripheral dentine wall thickness. This result was further supported by nonparametric Mann–Whitney U testing (*p* = 0.015). **Conclusions**: Cement layer thickness is a key biomechanical factor influencing the fracture resistance of endocrown restorations. Preparation depth and dentine wall geometry appear to have a less direct impact.

## 1. Introduction

Restoring endodontically treated teeth presents a clinical challenge, as these teeth often suffer from cumulative structural loss resulting from caries, access cavity preparation, and dentine removal during endodontic therapy. The reduced amount of sound dental tissue increases susceptibility to fracture and compromises long-term restorative success [1,2]. Traditionally, intraradicular posts—particularly fiber posts—have been advocated to enhance retention and improve stress distribution in severely compromised teeth [3,4]. However, clinical outcomes have shown that post placement alone does not consistently enhance fracture resistance, and its effectiveness is highly dependent on factors such as bonding interface quality and the remaining coronal structure [5,6,7]. Furthermore, current evidence suggests that the presence of a ferrule has a more substantial influence on biomechanical behavior than post selection alone [3,4,8].

In situations where achieving a sufficient ferrule is not feasible, alternative procedures such as surgical crown lengthening or orthodontic extrusion are often employed, but these approaches may unfavorably affect the crown-to-root ratio and overall tooth prognosis [9,10,11]. These limitations have led to the increasing adoption of endocrowns—restorations that offer a conservative, adhesive-based alternative by utilizing the pulp chamber for macromechanical retention—thereby eliminating the need for post placement and ferrule preparation.

Initially introduced by Pissis [12] and later formalized by Bindl and Mörmann [13], the endocrown concept has gained popularity for its simplicity, structural preservation, and favorable mechanical behavior. Advances in digital workflows and CAD/CAM technologies have further contributed to the success of endocrowns, enabling improved precision, internal adaptation, and esthetic integration [14,15,16,17,18]. These factors, combined with efficient adhesive protocols, make endocrowns a viable option for restoring root-filled posterior teeth. However, their long-term performance is influenced by critical preparation-related parameters, such as intracoronal cavity depth, dentine wall thickness, and cement layer thickness—all of which warrant careful biomechanical consideration.

Among these parameters, intracoronal depth determines the available bonding surface and has been associated with improved mechanical retention and fracture resistance, although some studies report diminishing returns beyond a certain threshold [19,20,21,22]. Similarly, dentine wall thickness, particularly in the lateral walls of the pulp chamber, has been proposed as a structural reinforcement component, yet its isolated effect remains inconclusive [23]. In addition, recent studies have underscored the relevance of cement layer thickness to adhesive restorations. Excessively thick cement layers may reduce bond strength due to polymerization shrinkage and stress concentration at the adhesive interface [24,25]. Although often overlooked, this parameter may significantly impact the mechanical performance of endocrowns, especially when the thickness exceeds thresholds of 200–300 µm [26,27].

Despite growing research interest in these biomechanical variables, few studies have simultaneously assessed the combined impact of intracoronal depth, dentine geometry, and cement thickness on endocrown performance. A comprehensive understanding of their interaction is essential to developing evidence-based guidelines for endocrown preparation.

In this study, SprintRay Crown^TM^ (SprintRay Inc., Los Angeles, CA, USA), a 3D-printed resin material, was selected for the fabrication of endocrowns due to its favorable mechanical properties, digital workflow compatibility, and cost-effectiveness. Resin-based CAD/CAM materials have demonstrated sufficient mechanical performance for posterior restorations and offer advantages in terms of internal adaptation and chairside production. Awada and Nathanson [17] reported that such materials combine the resilience of polymers with the esthetics and wear resistance of ceramics, making them suitable for load-bearing posterior applications.

Various 3D printing technologies have been applied in dentistry, including vat photopolymerization methods such as stereolithography (SLA), digital light processing (DLP), and masked stereolithography (MSLA), as well as material extrusion techniques like fused deposition modeling (FDM) [28]. These methods differ in terms of light source, resolution, and material compatibility. In the present study, endocrowns were printed using MSLA technology on a Prusa SL1 printer, which employs a masked LCD screen to cure photopolymer resin layer by layer.

The null hypothesis of this study was that intracoronal depth, dentine wall thickness, and cement layer thickness have no significant effect on the fracture resistance of molars restored with endocrowns. Accordingly, the aim of this study was to assess the individual and combined influence of these variables to provide guidance for optimal endocrown preparation protocols.

## 2. Materials and Methods

### 2.1. Tooth Selection and Endodontic Treatment

Twelve human molars that had been extracted due to periodontal disease were selected for this study. Teeth were included if they had no extensive structural damage, fractures, or significant coronal destruction. The selection criteria ensured a sufficient pulp chamber depth (≥ 2 mm) and the absence of calcifications, making them suitable for standardized endocrown preparation. Some teeth exhibited moderate coronal caries, reflecting typical clinical scenarios in which molars requiring endodontic treatment often present with partial structural loss.

This investigation was conducted as a pilot in vitro study, designed to explore biomechanical trends related to endocrown preparation variables. The sample size of 12 molars was chosen based on feasibility constraints and followed the precedent of similar laboratory studies on the fracture resistance of endodontically treated teeth. For example, Dartora et al. [19] used comparable group sizes to evaluate the effect of intracoronal depth on endocrown performance. Although not statistically powered for clinical generalization, the findings of this pilot study offer foundational data to support future studies with expanded sample sizes and comparative material analyses.

Similar feasibility-based designs have been employed in the literature. For instance, Abduljawad and Rayyan [29] conducted an endocrown study with 10 samples per group, citing sample size estimates based on prior studies and ANOVA-based power assumptions (80–90% power, α = 0.05, and β = 0.2). Turker Kader et al. [30] similarly used only four samples per group during their pilot protocol development phase. In addition, Altinci et al. [31] recently investigated the trueness and fit of additively manufactured endocrowns using a pilot sample size of four specimens per subgroup. These studies support the appropriateness of small-sample pilot models in in vitro testing for method validation and early trend detection.

Access cavities were carefully created using high-speed round diamond burs with water cooling. Canal negotiation was achieved using manual K-files size 10 (Kendo, VDW GmbH, Munich, Germany), and a glide path was achieved using a Proglider file (Dentsply Sirona, Charlotte, NC, USA). Preparation of root canals was performed using X1 and X2 rotary files from the Protaper Next Rotary system (Dentsply Sirona, Charlotte, NC, USA). The rotary files were operated using the manufacturer’s recommended speed and torque, namely, 300 rpm and 250 gcm. During the shaping process, the canals were irrigated with 5.25% sodium hypochlorite (NaOCl) and EDTA solution to ensure that patency was permanently achieved.

Root canal obturation was performed using the single cone technique with gutta-percha cones and ADSEAL (MetaBiomed, Cheongju, Republic of Korea) sealer. The gutta-percha cones were then seared at the canal orifices using a heated electronic plugger (Woodpecker Fi-P, Woodpecker, Guilin, China) and compacted vertically. The canal orifices and pulp chamber floor were then covered with SDR^®^ flow+ Bulk Fill Flowable (Dentsply Sirona, Charlotte, NC, USA).

This study was approved by the Scientific Research Ethics Committee of the ‘Victor Babeș’ University of Medicine and Pharmacy of Timișoara (UMFVBT), under Approval No. 37/04.12.2023.

### 2.2. Endocrown Preparation and Fabrication

For the endocrown preparation, butt joint margins were established. The intracoronal depth of the pulp chamber varied among samples and was measured individually using a periodontal probe. Since the pulp chamber floor was covered with SDR^®^ flow+ Bulk Fill Flowable after canal obturation, this composite surface served as the reference point for measuring and standardizing intracoronal cavity depth. This depth was clinically evaluated using a periodontal probe, and CBCT measurements confirmed a global mean depth of 3.05 mm across all specimens. All undercuts were carefully eliminated, and the preparation was refined using a yellow-coded cylindrical bur.

**Figure 1:**
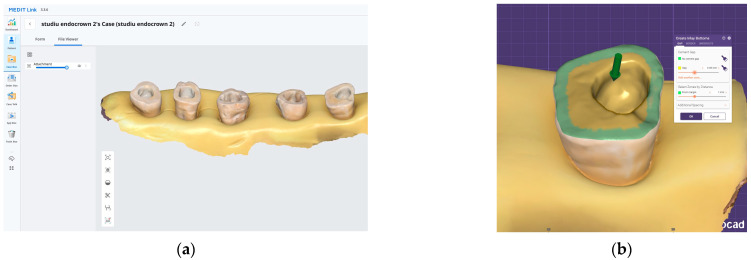
The digital workflow used for endocrown fabrication: (**a**) 3D scan of the prepared teeth, which are stabilized in a silicone base. (**b**) Exocad design interface illustrating the internal adaptation spacing of the endocrowns.

After preparation, each specimen was embedded in a silicone base to ensure stable positioning during scanning. The teeth were scanned using the Medit i700 intraoral scanner (Medit, Seoul, Republic of Korea) (Figure 1a). All intraoral scans were performed by the same operator using a standardized acquisition protocol.

All restorations were designed in Exocad software (Version 3.0, Exocad GmbH, Darmstadt, Germany) with a standardized cement gap of 0.1 mm, except for a 0.6 mm zone above the restoration margin where no spacer was applied (intimate adaptation) (Figure 1b). This virtual spacer setting was applied uniformly across all samples as a controlled internal fit parameter.

After design completion, the restorations were exported for printing using SprintRay Crown^TM^ (SprintRay Inc., Los Angeles, CA, USA), a resin-based permanent restorative material. The prints were fabricated via masked stereolithography (MSLA) on a Prusa SL1 3D printer (Futur3D, Prague, Czech Republic), then they were post-processed, sandblasted, and glazed. To minimize variability and isolate the effects of the preparation-related parameters, all other factors—material type, scanner, printer, software, operator, cementation procedure, and testing protocol—were standardized across all samples.

### 2.3. CBCT Imaging and Measurement

**Figure 2:**
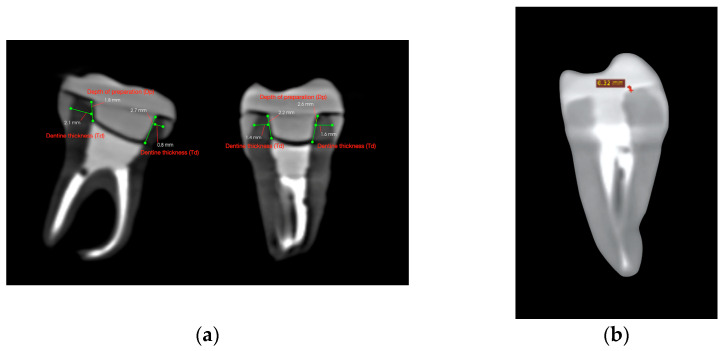
CBCT cross-sectional images used for volumetric assessment. (**a**) Pre-cementation view: mesiodistal and buccolingual CBCT cross-sections used for dentine thickness and intracoronal depth measurement. (**b**) Post-cementation view: CBCT image illustrating cement layer thickness at the dentine–restoration interface.

After endocrown preparation, the teeth were securely placed in a polystyrene support in an arched pattern, to replicate a dental arch. A cone-beam computed tomography (CBCT) scan was performed prior to cementation to ensure accurate measurement of dentine thickness, intracoronal depth, and occlusal height. Scans were conducted using the Carestream CS 9600 system (Carestream Dental LLC, Atlanta, GA, USA), ensuring high-resolution imaging (Figure 2a).

**Figure 3:**
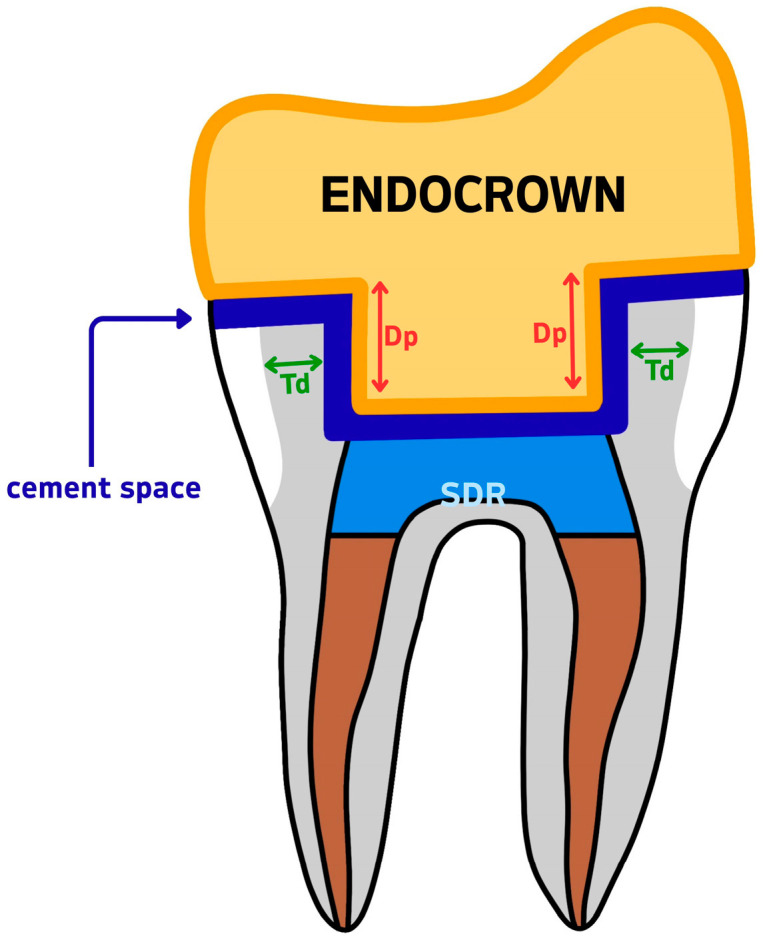
Measurement reference diagram for CBCT-based variables. Td = dentine wall thickness; Dp = intracoronal depth of the pulp chamber extension; SDR = Smart Dentin Replacement flowable composite; cement space = the virtual internal gap filled with resin cement.

Each tooth was cross-sectioned and measured in two planes: the mesio-distal and the bucco-lingual. In each section, dentine thickness (Td) was measured at the middle of the vertical axial wall—mesially and distally in the mesio-distal section, and buccally and lingually in the bucco-lingual section. Similarly, the endocrown pulp chamber extension (Dp) was measured twice per section. The measurement sites are illustrated in Figure 3.

In total, four measurements were taken per tooth for dentine thickness and four for intracoronal depth. The mean values per tooth were calculated for reference purposes. However, the statistical analysis was performed using the minimum recorded values for each variable, as described in Section 2.6. Occlusal height (Ho) was measured using the same CBCT cross-sectional images and recorded in millimeters using the linear measurement tool in CS 3D imaging software, version 3.46.10 (Carestream Dental LLC, Atlanta, GA, USA). Cement thickness (Tc) was assessed using a second CBCT scan performed after cementation, with the same scanning parameters as the initial scan. For each specimen, Tc was measured at nine evenly spaced points along a single coronal cross-sectional plane, selected to represent the adhesive interface near the butt-joint margin between the tooth and the endocrown. This strategy enabled standardized sampling of the cement layer at the horizontal marginal interface. The mean of these values was calculated per tooth and used for statistical analysis, as it offers a more representative estimate of average interfacial adaptation. The minimum, maximum, and mean values recorded for each specimen are reported in Table 1.

**Figure 4:**
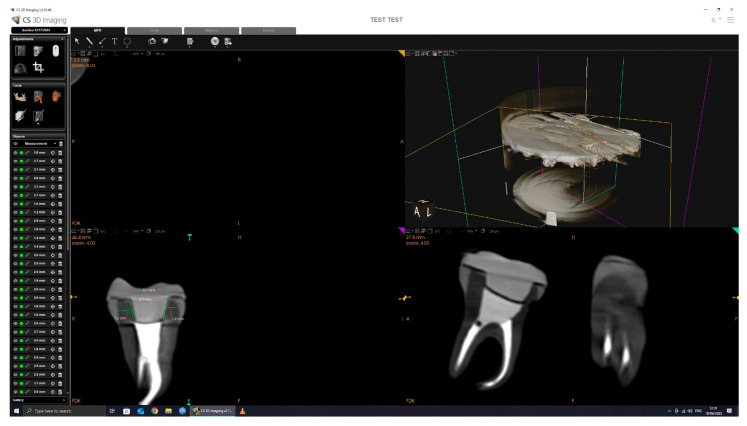
CBCT scan and measurement.

The CBCT scan parameters were set to 90.00 kV, 4.00 mA, and 20.00 s exposure, with a dose of 1140.48 mGy/cm^2^. The 3D images were processed and analyzed using CS 3D Imaging software version 3.46.10 (Carestream Dental LLC, Atlanta, GA, USA), with a slice thickness of 150 µm (Figure 4).

All dimensional measurements were performed using the linear caliper tool in CS 3D Imaging software, version 3.46.10 (Carestream Dental), which is internally calibrated according to the known voxel size (150 µm) of the scan. Measurements were taken in calibrated axial cross-sections, and all data collection was conducted by the same operator to ensure consistency. All CBCT scans were conducted by the same operator using a standardized protocol. Tooth and crown positioning was consistently performed using the same polystyrene arch support and placement technique across all samples to ensure reproducibility. This approach minimized variability in image orientation and measurement accuracy.

### 2.4. Adhesive Cementation

The endocrowns were polished, ultrasonically cleaned, and sandblasted with 50 μm alumina particles, then thoroughly cleaned using anhydrous alcohol and dried with oil-free air.

Scotchbond Universal Plus Adhesive (3M, Maplewood, MN, USA) was applied by actively scrubbing for 20 s onto the internal surface of the endocrown, followed by gentle air-drying for 5 s using oil-free air to evaporate the solvent. The adhesive was not light-cured prior to seating, in accordance with the manufacturer’s co-curing protocol when used in combination with RelyX Universal Resin Cement (3M, Maplewood, MN, USA).

Selective etching was performed on the enamel margins, using 37% phosphoric acid for 15 s, followed by thorough rinsing with distilled water for 30 s and gentle drying with oil-free air. Scotchbond Universal Plus Adhesive (3M, Maplewood, MN, USA) was then applied to both enamel and dentine surfaces and gently air-dried.

Relyx Universal Resin Cement (3M, Maplewood, MN, USA) was applied to the intaglio surface of the endocrowns, which was then gently placed in position on the tooth preparation. Tack curing was performed with the LED-E Woodpecker (Woodpecker, Guilin, China), and any excess cement was removed. Final light-curing was performed for 10 s per axial surface using a high-intensity LED curing light (>1200 mW/cm^2^), as recommended by the manufacturer. Where light access was limited, chemical polymerization was allowed to proceed for a minimum of 6 min.

### 2.5. Fracture Resistance Testing

**Figure 5:**
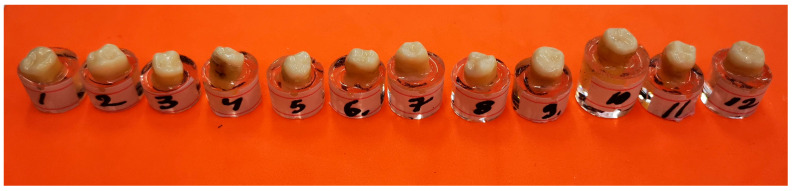
Specimens placed in epoxy resin.

After the endocrowns were adhesively cemented, the specimens were prepared for mechanical testing. Each specimen was placed in an epoxy resin support (Epodex, Krefeld, Germany) (Figure 5).

To evaluate the fracture resistance of the restored molars, a compressive load was applied using a universal testing machine (LBG TC100, LBG, Azzano s. Paolo, Italy). The machine was equipped with a 50 kN force cell (0.001 kN resolution), and the loading speed was set to 2 mm/min. The test was stopped when a sudden drop in force below 80% of the maximum recorded load occurred. The load was applied vertically, aligned with the long axis of each specimen, using a 5 mm diameter spherical metal indenter centered in the occlusal groove. While 1 mm/min is frequently used in fracture resistance tests, a crosshead speed of 2 mm/min was chosen as a practical compromise to ensure quasi-static loading while maintaining operational efficiency. This approach allows for the precise identification of fracture initiation under clinically relevant compressive conditions.

**Figure 6:**
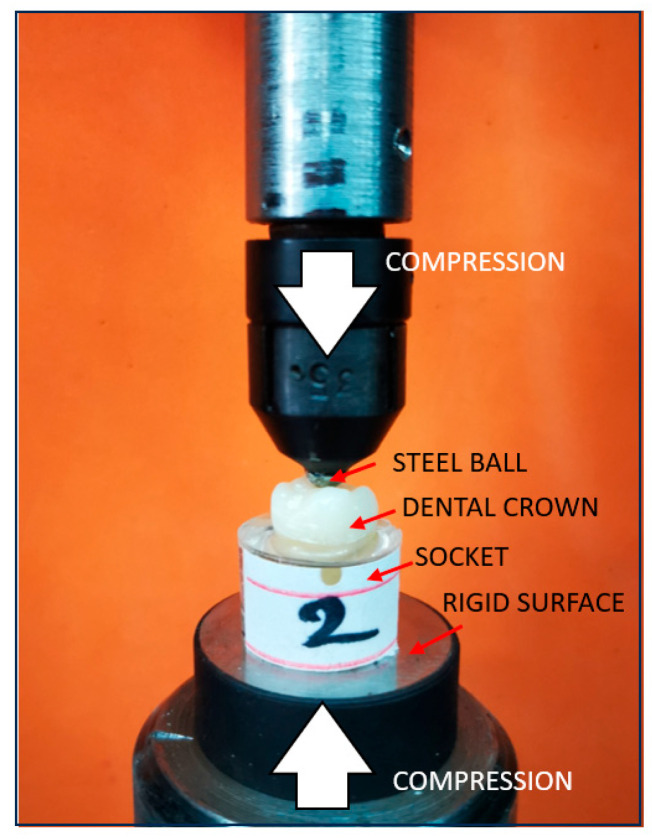
Mechanical testing of the endocrown.

Each sample (*n* = 12) was loaded in a configuration consisting of a flat surface (lower support) with a spherical metal indenter (5 mm diameter) positioned along the longitudinal axis of the tooth, centered in the occlusal groove (Figure 6). The aim of this procedure was to determine the maximum compressive force required to fracture the cemented endocrowns, along with the corresponding displacement and energy absorbed until failure.

The data were also evaluated from a statistical perspective. The normality of the recorded data was verified using the Kolmogorov–Smirnov and Shapiro–Wilk tests, confirming that force, displacement, and energy data followed a normal distribution. The results of the statistical analysis will be presented in the following section.

**Figure 7:**
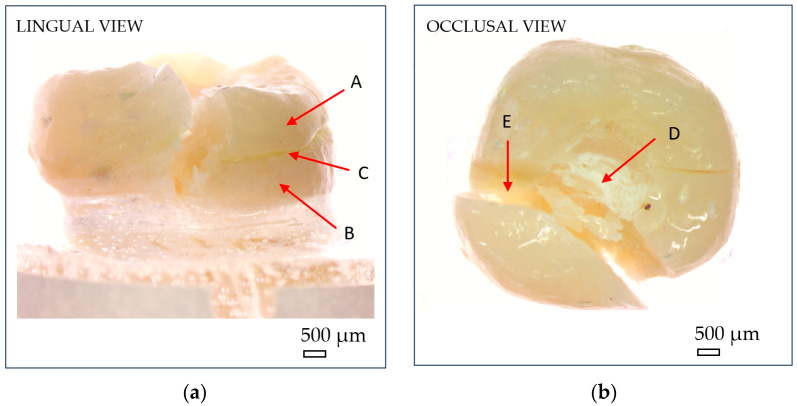
Optical microscopy images showing macroscopic fracture features of endocrowns: (**a**) lingual view; (**b**) occlusal view. (A) reconstructed crown; (B) natural tooth; (C) luting cement; (D) compressive damage zone at the indenter–crown interface; (E) fracture zone. Scale bar = 500 µm.

**Figure 8:**
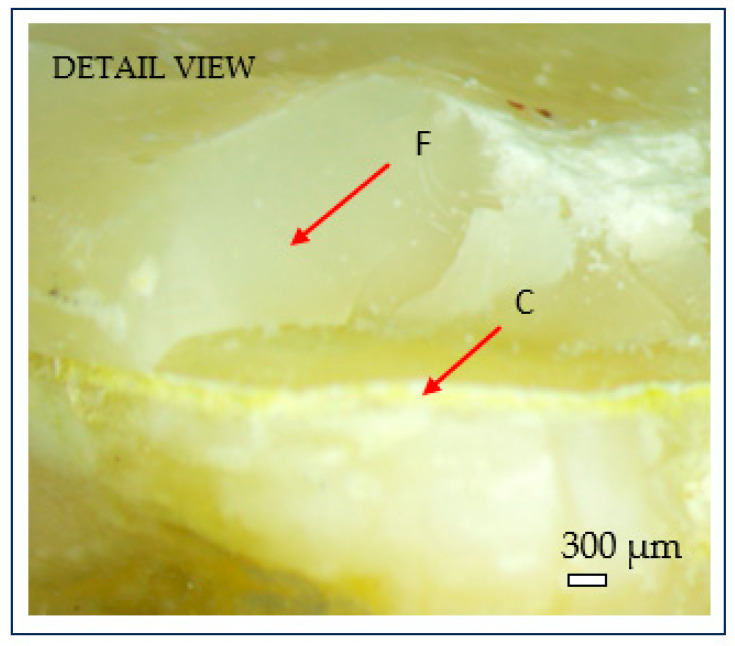
Microscopic detail of brittle fracture at the dentine–cement interface: (C) cement layer; (F) brittle fracture surface. Scale bar = 300 µm.

After fracture, the failure mode of each sample was inspected using an optical microscope (Optika SLX-3, OPTIKA S.r.l., Ponteranica, Italy) at 10× magnification. Crack initiation was typically observed at the occlusal contact area with the metal indenter, originating from surface crushing and then propagating longitudinally and transversely within the tooth structure (Figure 7). Fracture patterns and failure characteristics were documented and classified accordingly (Figure 8).

### 2.6. Statistical Analysis

Data were analyzed using IBM SPSS Statistics version 26 (IBM Corp., Armonk, NY, USA). For each tooth, mean values were calculated for dentine thickness and endocrown pulp chamber depth from four CBCT measurements. However, for statistical correlation and group comparison analyses, the minimum recorded value per variable was used per tooth, based on the mechanical rationale that structural failure typically occurs at the weakest point.

Pearson correlation coefficients were calculated to assess the strength and direction of relationships between the independent variables (Td, Dp, Tc, Ho) and the dependent variable (fracture force, FF).

A one-way analysis of variance (ANOVA) was applied to evaluate differences in fracture force between groups defined by structural thresholds.

Grouping thresholds for each variable were determined based on the distribution of observed values in the dataset. Specimens were divided into two groups (*n* = 6) per variable (Td, Dp, Tc). For dentine thickness (Td) and depth of preparation (Dp), grouping was based on the minimum recorded value per tooth, reflecting the rationale that failure typically initiates at the weakest point. Cement thickness (Tc) was grouped based on the mean of nine CBCT measurements per tooth, to better represent average adhesive interface adaptation.

Detailed directional measurements for Td, Dp, and Tc, along with fracture force values, are provided in Supplementary Table S1.

The following group thresholds were used:

- Td: Group 1 < 1 mm; Group 2 ≥ 1 mm
- Dp: Group 1 < 2.5 mm; Group 2 ≥ 2.5 mm
- Tc: Group 1 < 0.3 mm; Group 2 ≥ 0.3 mm

For post hoc comparison and significance testing, the F-statistic and corresponding *p*-values were interpreted at a significance level of *p* < 0.05.

To account for the small sample size and potential deviations from normality, a nonparametric Mann–Whitney U test was also performed to confirm the consistency of the ANOVA results.

## 3. Results

### 3.1. Mechanical Testing Results

Before proceeding to the variable-based statistical analysis, the raw mechanical testing outcomes were evaluated. The fracture resistance of the restored molars was assessed using a universal testing machine, with each specimen subjected to compressive loading until catastrophic failure.

**Figure 9:**
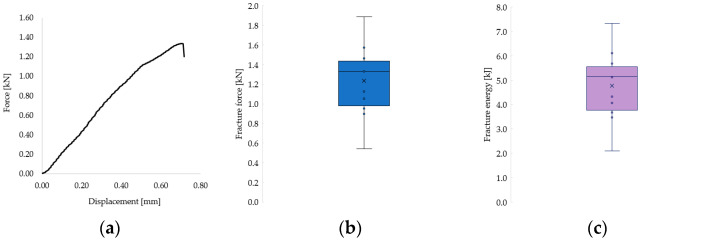
(**a**) Representative force–displacement curve showing the brittle fracture behavior of a 3D-printed endocrown specimen. (**b**) Distribution of cement layer thickness across all tested samples. (**c**) Fracture resistance values grouped according to cement thickness categories.

The recorded fracture forces ranged from 543 N to 1889 N, while the energy absorbed until failure (calculated as the area under the force–displacement curve) ranged from 2.10 kJ to 7.34 kJ. The mechanical behavior of the restorations revealed a predominantly brittle failure pattern, characterized by a sudden drop in force without a plastic deformation plateau, as illustrated in the representative force–displacement curve (Figure 9a) and confirmed by microscopic observations (Figure 8).

The distribution of individual fracture force and energy values across all samples is shown in Figure 9b and Figure 9c, respectively. Most values clustered in the second quartile, with notable variability that is likely attributable to inherent structural differences between natural teeth. Given the complexity of the failure surfaces, fracture stress could not be calculated, as it was not feasible to determine the precise fracture area.

Statistical analysis was conducted to evaluate the relationship between dentine thickness (Td), depth of preparation (Dp), occlusal height (Ho), and cement thickness (Tc) as independent variables, with fracture force (FF) as the dependent variable. Pearson correlation and a one-way ANOVA were used to determine the strength and significance of these associations. All independent variables were measured in millimeters (mm), and fracture force (FF) was recorded in newtons (N).

### 3.2. Descriptive Statistics

Descriptive statistics were used to evaluate fracture force (FF) across the subgroups formed according to threshold values for dentine thickness (Td), depth of preparation (Dp), and cement thickness (Tc). The group with higher cement thickness (Tc ≥ 0.3 mm) showed the highest average fracture force (1424.84 N), while the group with lower Tc values (< 0.3 mm) showed a considerably lower average of 973.25 N. Regarding dentine thickness and depth of preparation, the differences in mean fracture force between the groups were less pronounced (Table 2). The individual minimum, maximum, and mean values for each variable per sample are detailed in Table 1, providing a comprehensive overview of the measured CBCT-based parameters and fracture resistance outcomes.

### 3.3. Correlation Analysis

Pearson’s correlation coefficients were calculated to assess the strength and direction of the relationships between the independent variables and fracture force. The highest correlation was observed between cement thickness (Tc) and fracture force (FF), with a positive coefficient of r = 0.577 indicating a strong association. Dentine thickness (Td) and occlusal height (Ho) showed weaker positive correlations (r = 0.210 and r = 0.113, respectively), while depth of preparation (Dp) was moderately negatively correlated with FF (r = −0.390), suggesting that deeper cavities may reduce fracture resistance (Table 3).

### 3.4. ANOVA Analysis

A one-way ANOVA was used to compare fracture force (FF) between groups defined by threshold values of Td (1 mm), Dp (2.5 mm), and Tc (0.3 mm). Statistically significant differences were found only in the case of cement thickness. The ANOVA for Tc yielded an F-value of 7.847 and a *p*-value of 0.019, indicating a significant effect of cement thickness on fracture resistance. In contrast, dentine thickness (F = 1.042; *p* = 0.331) and depth of preparation (F = 0.016; *p* = 0.902) did not show significant differences between groups (Table 4).

These results confirm that cement thickness is the only independent variable that significantly influences fracture force in endocrown restorations, which is consistent with the observed fracture patterns at the dentine–cement interface.

While most studies on endocrowns have focused on milled ceramic restorations such as lithium disilicate, 3D-printed resin-based materials offer a more cost-effective and accessible alternative for clinical use. These materials can be fabricated with high precision using additive manufacturing workflows. Despite their growing popularity, limited data exist on the biomechanical performance of 3D-printed endocrowns, particularly in molars subjected to high occlusal forces. Accordingly, this study sought to evaluate the fracture resistance of 3D-printed resin-based endocrowns and investigate how preparation-related variables influence their mechanical behavior.

The null hypothesis of this study stated that intracoronal depth, dentine wall thickness, and cement layer thickness would have no significant effect on the fracture resistance of endocrown restorations. Based on the statistical results, this hypothesis was partially rejected. Cement thickness was found to have a statistically significant influence on fracture resistance (ANOVA F = 7.847, *p* = 0.019), whereas dentine thickness and intracoronal depth did not demonstrate significant associations. These findings indicate that cement layer thickness is a critical factor influencing mechanical performance, while the effects of intracoronal depth and dentine wall thickness may be less direct or context-dependent.

Due to the small sample size, a nonparametric analysis (Mann–Whitney U test) was also performed, which confirmed the same statistical outcome as the ANOVA: a significant difference in fracture force was found only for the cement thickness (Tc) grouping.

A side-by-side comparison of the ANOVA and Mann–Whitney U test results is provided in Table 5 to illustrate the consistency of findings across statistical methods.

## 4. Discussion

### 4.1. Interpretation of Findings

The findings of this study underscore the central role of cement layer thickness in determining the fracture resistance of endocrowns, with intracoronal depth and dentine wall thickness demonstrating a less direct correlation. These results are consistent with the current literature highlighting the biomechanical relevance of luting agent thickness in adhesive restorations.

May et al. [24], Rojpaibool and Leevailoj [26], and Tribst et al. [27] all reported that excessive cement thickness contributes to stress concentration, impairs polymerization efficiency, and ultimately reduces both bond strength and fracture resistance. Our data support these findings, as cement thickness was the only variable to show statistical significance in the ANOVA model (F = 7.847, *p* = 0.019) and demonstrated a strong positive correlation with fracture resistance (r = 0.577). Given the limited sample size, a nonparametric Mann–Whitney U test was performed to support the reliability of the findings. The test confirmed a statistically significant difference in fracture force between cement thickness groups (*p* = 0.015), which was in line with the ANOVA results.

The implication is that a well-distributed, appropriately polymerized adhesive interface significantly enhances the structural integrity of endocrowns under functional load. May et al. [24] also achieved superior performance when a 25 µm cement space was used, reinforcing the importance of internal space design precision in CAD/CAM workflows.

Although our study utilized a resin-based material (SprintRay Crown^TM^), similar trends regarding cement thickness have been reported in lithium disilicate restorations. Rojpaibool and Leevailoj [26] found that cement layers exceeding 200 µm negatively affected fracture resistance in ceramic endocrowns, supporting the threshold effect suggested by our findings.

Cement thickness appears to exert a more pronounced influence on biomechanical outcomes than the adhesive system itself. Sedrez-Porto et al. [32] and Rocca et al. [25] noted that fracture resistance in endocrowns was more affected by material choice, marginal adaptation, and preparation design than by the specific bonding protocol used. This interpretation is reinforced by Keskin et al. [33], who showed that fully digital workflows significantly improved internal adaptation, directly impacting cement thickness and overall mechanical performance.

Although parametric tests such as the Pearson correlation and ANOVA were applied, the small sample size may limit the robustness of distributional assumptions such as normality and homogeneity of variance. These tests were chosen in line with similar pilot in vitro studies, but their findings should be interpreted cautiously. This limitation reinforces the exploratory scope of the current investigation and points to the need for confirmatory research using larger sample sizes and alternative statistical approaches.

Polymerization dynamics further complicate the interaction between material properties and restoration geometry. Ikemoto et al. [34] evaluated the impact of CAD/CAM material thickness and translucency on the polymerization of dual-cure resin cement in endocrown restorations and confirmed that greater ceramic thicknesses reduce light transmission and impair polymerization effectiveness. These findings further emphasize the need to optimize both cement thickness and restorative design to ensure adequate polymerization.

In contrast to cement thickness, intracoronal depth in this study showed a weak or nonlinear correlation with fracture resistance. While Dartora et al. [19] reported significantly higher fracture resistance with a 5 mm intracoronal extension compared to shallower designs, other studies have produced less consistent results. Hayes et al. [20] and Zhang et al. [21] found that increasing preparation depth did not always improve mechanical performance and could even increase stress concentrations in the remaining tooth structure. Finite element analyses further support the idea that, while moderate depth improves stress distribution, excessive cavity depth may concentrate stresses at the dentine–cement interface or root walls [19,21]. In line with these findings, Saker et al. [22] evaluated endocrowns with varying pulpal extension depths and ferrule configurations. Their results indicated that while deeper extensions can increase the adhesion surface and may improve stress distribution, this did not consistently enhance fracture resistance in conventional endocrowns without ferrule design. In fact, fracture resistance values slightly decreased at 4 mm depth compared to 3 mm, and most failures occurred within the tooth structure itself, highlighting a potential weakening of the dentine and the importance of preserving tooth integrity. Collectively, these studies underscore the need to balance intracoronal extension depth with the preservation of a healthy tooth structure to optimize mechanical performance without compromising dentine integrity.

The impact of dentine wall thickness also warrants consideration. Although our data did not show a statistically significant correlation between lateral dentine thickness and fracture resistance, its potential biomechanical relevance has been discussed in recent studies. Li et al. [23] found no significant difference in fracture resistance across varying pulp chamber wall thicknesses but acknowledged that debonding remains a primary mode of failure in endocrown restorations. Dartora et al. [19] similarly emphasized the structural reinforcing role of peripheral dentine walls, with finite element analysis (FEA) indicating that thicker dentine reduces interfacial stress concentrations and improves load distribution.

While our findings did not confirm a direct linear relationship between dentine thickness and fracture resistance, the literature supports its contribution to overall biomechanical stability. Preserving dentine walls during preparation may, therefore, play a critical role in minimizing adhesive stress and reducing the risk of catastrophic failure.

Taken together, the findings from this study and the supporting literature indicate that cement layer thickness is not only a determinant of adhesive quality but also a primary predictor of endocrown fracture resistance. Depth of preparation and dentine wall geometry, while less statistically impactful in this study, should still be considered integral components of the biomechanical framework of endocrowns. Future studies should investigate the interaction of these parameters in larger sample sizes and explore advanced modeling approaches to better characterize nonlinear biomechanical behavior.

### 4.2. Clinical Implications

The clinical relevance of these findings lies in the optimization of adhesive protocols and restorative preparation strategies to enhance the mechanical performance of endocrowns. Given that cement thickness was identified as the primary determinant of fracture resistance, clinicians should prioritize achieving a thin, uniform, and well-polymerized luting layer. This entails a precise internal fit of CAD/CAM endocrowns, careful control of the virtual cement gap during design, and consideration of cement viscosity and application method.

Moreover, the findings highlight the importance of using light-transmissible ceramic materials and high-performance dual-cure resin cement to overcome polymerization limitations under thick restorations. Clinicians should be cautious when planning deep endocrown preparations, as excessive intracoronal depth does not necessarily translate into improved mechanical strength and may compromise adhesive curing. As discussed above (Section 4.1), previous studies suggest that intracoronal depths beyond 3.5–4 mm may offer diminishing mechanical returns and may even increase stress concentration in the cavity walls.

Although dentine wall thickness was not statistically significant in our study, preserving sufficient peripheral dentine during preparation remains biomechanically prudent. It contributes to the structural integrity of the tooth–restoration complex and may play a role in long-term performance.

Ultimately, individualized treatment planning that accounts for tooth anatomy, restoration geometry, and adhesive materials will allow clinicians to enhance the longevity and reliability of endocrown restorations. These results underscore the need for meticulous restorative protocols, especially in posterior teeth subjected to high occlusal loads.

### 4.3. Study Limitations

Despite the informative results obtained, this study presents certain limitations that must be acknowledged. First, this study utilized a standardized sample of 12 extracted human molars, which, although representative, may still limit the generalizability of the findings across broader clinical scenarios. Variability in tooth anatomy, occlusal forces, and patient-specific factors in vivo cannot be fully simulated in an in vitro setup.

The absence of a formal power analysis is acknowledged as a limitation. However, the sample size selection follows a precedent set by other pilot studies in this field [29,30,31], and the present study provides foundational effect-size data for future power calculations.

Second, while the fracture resistance testing protocol in this study followed standardized in vitro procedures, it did not incorporate the additional simulation of long-term intraoral usage conditions such as thermomechanical cycling or fatigue loading. Although these extended protocols are not routinely required in laboratory fracture testing, they may better reflect clinical aging processes and influence long-term restoration performance.

Additionally, the cementation protocol was standardized in this study; however, the influence of different resin cement types, adhesive systems, and light-curing protocols was not independently evaluated. While this study highlighted the role of cement thickness, future investigations could isolate and compare the influence of luting agent chemistry and application techniques.

Finally, although CBCT imaging with a voxel size of 150 μm provided a valuable quantitative assessment of dentine wall thickness and intracoronal depth, minor variability in image resolution and software calibration could affect dimensional accuracy. Micro-CT, which offers superior resolution when analyzing internal microstructures, was not available during the course of this study. Therefore, CBCT was used as an accessible and clinically relevant alternative. Moreover, the measurement of cement layer thickness using CBCT may also present limitations, due to the voxel size approaching the lower limit of the feature being measured (e.g., 40–80 µm) and the inherent contrast resolution of the imaging system, which could affect the precision of thin layer detection and segmentation during analysis.

Furthermore, while this study employed statistically robust methods, potential nonlinear or synergistic interactions between variables may require more advanced modeling approaches in future research, such as multivariate regression or finite element simulations.

### 4.4. Future Directions

Building on the current findings, future research should aim to expand our understanding of the biomechanical factors influencing endocrown performance through more comprehensive and clinically relevant methodologies. Studies incorporating larger sample sizes and including a broader range of tooth types and morphologies would enhance the study’s statistical power and improve the generalizability of results.

It is also recommended that future investigations integrate thermomechanical aging, cyclic loading, and long-term fatigue testing to simulate intraoral conditions more accurately. These protocols would allow for a better assessment of adhesive interface durability and the behavior of restorations under functional stresses over time.

Further studies should evaluate the role of varying adhesive strategies, luting agents, and cement viscosities in relation to cement thickness and restoration fit. Comparative assessments between self-adhesive, etch-and-rinse, and universal adhesive systems—especially in combination with different cementation protocols—would help identify the most effective bonding approaches under different clinical conditions.

In addition, advanced imaging techniques, such as high-resolution micro-CT or optical coherence tomography, may be employed in future studies to improve the precision of cement layer and internal fit measurements beyond the level that CBCT currently allows. Finite element analysis (FEA) modeling can also be used to explore stress distribution patterns and material behavior in more complex geometries and under varied loading conditions.

Finally, longitudinal clinical studies and randomized controlled trials are needed to validate in vitro findings and translate the laboratory findings into clinical relevance and real-world treatment outcomes. Assessing restoration longevity, failure modes, and patient-reported outcomes over time will provide more robust evidence for clinical decision-making and treatment planning involving endocrowns.

## 5. Conclusions

Within the limitations of this pilot in vitro study, the following exploratory observations were noted:

- Cement thickness showed a trend toward a positive correlation with fracture resistance in 3D-printed endocrowns.
- Dentine wall thickness and intracoronal cavity depth appeared to have a less pronounced biomechanical impact.
- Fractures frequently began at the cement–dentine interface, highlighting the role of adhesive integrity.

These preliminary findings suggest areas for future investigation, but these should not be interpreted as clinical recommendations. Further research with larger sample sizes and long-term simulations is required before clinical application can be considered.

## Figures and Tables

**Table 1:** Summary of CBCT-based measurements and fracture force per sample.

Sample	Td (mm)			Dp (mm)			Tc (mm)			FF (N)
	**Min**	**Max**	**Mean**	**Min**	**Max**	**Mean**	**Min**	**Max**	**Mean**	
1	1.2	1.9	1.53	1.6	4.1	3.2	0.31	0.46	0.35	1889.154
2	1	2.3	1.78	2.5	3.3	2.88	0.23	0.48	0.36	1331.296
3	0.6	2.2	1.6	2.5	3.1	2.9	0.2	0.32	0.26	1123.978
4	1.3	1.7	1.7	3	4.8	4.25	0.22	0.28	0.25	952.6722
5	1.1	1.9	1.35	2.8	3.2	2.98	0.29	0.38	0.32	1332.121
6	0.5	1.3	1	2.4	3	2.63	0.19	0.31	0.25	899.3144
7	0.9	2.2	1.3	2.5	2.6	2.55	0.24	0.35	0.30	1573.965
8	0.9	2.7	1.63	2.4	2.7	2.5	0.17	0.31	0.25	543.1567
9	0.8	1.6	1.48	1.8	2.7	2.33	0.36	0.47	0.39	1329.479
10	1.3	1.7	1.45	1.9	3.6	2.88	0.21	0.36	0.29	1347.155
11	1.6	2.5	1.95	2.6	2.9	2.68	0.26	0.32	0.28	1052.449
12	1.2	1.6	1.4	2.9	3.1	2.93	0.23	0.36	0.30	1465.43

**Table 2:** Descriptive statistics of fracture force (FF) per subgroup (in newtons, N).

Dependent Variable	Groups	Count	Sum	Mean	Variance
FF_Td_	gr.1	6	6801.18	1133.53	134,995.8
	gr.2	6	8038.98	1339.83	110,024.9
FF_Dp_	gr.1	6	7339.55	1223.25	209,840.1
	gr.2	6	7500.61	1250.10	60,283.79
FF_Tc_	gr.1	6	5918.72	986.45	71,724.08
	gr.2	6	9973.89	1486.91	4856.01

FFTd, FFDp, and FFTc refer to fracture force grouped by dentine thickness, depth of preparation, and cement thickness, respectively.

**Table 3:** Pearson correlation matrix between independent variables (Td, Dp, Ho, Tc) and fracture force (FF).

Variable	Td	Dp	Ho	Tc	FF
Td	1.000	0.131	0.117	0.253	0.210
Dp	0.131	1.000	0.164	−0.405	−0.390
Ho	0.117	0.164	1.000	0.249	0.113
Tc	0.253	−0.405	0.249	1.000	0.577
FF	0.210	−0.390	0.113	0.577	1.000

Td = dentine thickness; Dp = depth of preparation; Ho = occlusal height; Tc = cement thickness; FF = fracture force.

**Table 4:** One-way ANOVA results for fracture force (FF).

Source of Variation	SS	df	MS	F	*p*-Value	F Crit
FFTd	127,677.4	1	127,677.4	1.042	0.331	4.965
FFDp	2161.7	1	2161.7	0.016	0.902	4.965
FFTc	594,797.5	1	594,797.5	7.847	0.019	4.965

FF = fracture force; FFTd, FFDp, and FFTc = fracture force grouped by dentine thickness, depth of preparation, and cement thickness; SS = sum of squares; df = degrees of freedom; MS = mean square; F = F-statistic; F crit = critical F-value at α = 0.05.

**Table 5:** Comparison of fracture force values between subgroups for each structural parameter using parametric (ANOVA) and nonparametric (Mann–Whitney U) statistical tests.

Variable	Group Threshold (mm)	ANOVA (*p*-Value)	Mann–Whitney U (*p*-Value)
Td	< 1.0 mm vs. ≥ 1.0	0.331	0.310
Dp	< 2.5 mm vs. ≥ 2.5	0.902	0.818
Tc	< 0.30 mm vs. ≥ 0.30	0.019	0.015

## Data Availability

The raw data supporting the conclusions of this article will be made available by the authors on request.

## Supplementary Materials

The following supporting information can be downloaded at: https://www.mdpi.com/article/10.3390/dj13060263/s1. Table S1: Raw measurement data.
